# An Immunocompetent Host With Severe Disseminated Herpes Simplex Virus Type 2 Infection With Hepatorenal Involvement

**DOI:** 10.7759/cureus.95867

**Published:** 2025-10-31

**Authors:** Sehrish Baloch, Abdul Wasio, Bethel Shiferaw, Anoud Khan, Saqib Raza Khan

**Affiliations:** 1 Department of Oncology, Aga Khan University Hospital, Karachi, PAK; 2 Department of Internal Medicine, Saint Mary's Hospital, Waterbury, USA; 3 Department of Medicine, Ziauddin Medical College, Karachi, PAK; 4 Department of Oncology, Schulich School of Medicine and Dentistry, London, CAN; 5 Department of Oncology, Verspeeten Family Cancer Centre, London Health Sciences Centre, London, CAN

**Keywords:** acute kidney injury, disseminated infection, hepatitis, hsv2, immunocompetent host, liver injury

## Abstract

Herpes simplex virus type 2 (HSV-2) most commonly causes localized mucocutaneous infections, whereas disseminated disease with visceral involvement is rare, particularly in immunocompetent individuals. HSV hepatitis is a potentially life-threatening manifestation that often presents with nonspecific symptoms and markedly elevated liver enzymes, making early recognition difficult. We report a 58-year-old immunocompetent man with good performance status (Eastern Cooperative Oncology Group (ECOG) 0) who presented with fever, malaise, and a progressive vesicular rash involving the face, hands, and trunk. Laboratory investigations revealed elevated transaminases, hyperbilirubinemia, acute kidney injury, and electrolyte abnormalities. Imaging demonstrated mild hepatosplenomegaly without obstruction, and polymerase chain reaction testing confirmed HSV-2 in both serum and skin lesions. Other infectious and autoimmune evaluations were negative. The patient was treated with intravenous acyclovir followed by oral valacyclovir, resulting in complete resolution of symptoms and normalization of liver and renal function. Given ongoing high-risk sexual behavior, he was also counseled on long-acting injectable HIV pre-exposure prophylaxis with lenacapavir. The current case highlights that disseminated HSV-2 infection with hepatic and renal injury can occur in an immunocompetent host. Early recognition and prompt antiviral therapy are critical to improving clinical outcomes. Physicians should consider a high index of suspicion for HSV hepatitis in candidates presenting with unexplained transaminitis and systemic symptoms, even in the absence of classic mucocutaneous findings or immunosuppression.

## Introduction

Herpes simplex virus (HSV) infection remains highly prevalent in the United States. HSV-1 is commonly associated with oral lesions but also considered a cause of genital infections, affecting an estimated 50-80% of adults [1]. HSV-2, which primarily causes genital herpes, has a prevalence of approximately one in six individuals aged 14-49 years. Model-based estimates suggest that over 500,000 new genital HSV-2 infections occur annually in the United States. In comparison, HSV-1 may account for as many as three million new infections per year, including up to 500,000 new genital HSV-1 cases [2].

Disseminated HSV‑2 infection is uncommon in immunocompetent adults but has the potential to cause severe organ dysfunction, including hepatitis and renal impairment. Outside of the immunosuppressed population, HSV hepatitis is sporadic. It often presents with nonspecific systemic symptoms and markedly deranged liver enzymes [3-5]. Therefore, early diagnosis and appropriate management are crucial in preventing mortality.

We report an unusual case of HSV‑2 dissemination with hepatic and renal involvement in an immunocompetent patient, successfully treated with acyclovir, and he was also advised on long‑acting injectable HIV pre-exposure prophylaxis (PrEP) due to ongoing high-risk sexual behaviour.

## Case presentation

A 58-year-old man with a past medical history of coronary artery disease (CAD), hypertension, and hyperlipidemia presented with a one-week history of fever, chills, malaise, fatigue, and a progressive vesicular rash affecting his hands, face, and posterior trunk. The rash was vesicular in nature, initially appearing on the hands and later spreading to the face, abdomen, and posterior trunk. At presentation, it was disseminated, while the mucous membranes, eyes, and genitals were spared. He denied any history of asthma, eczema, recent medications, or tick bites. He recently travelled to California and reported unprotected sexual contact with multiple male partners.

**Figure 1:**
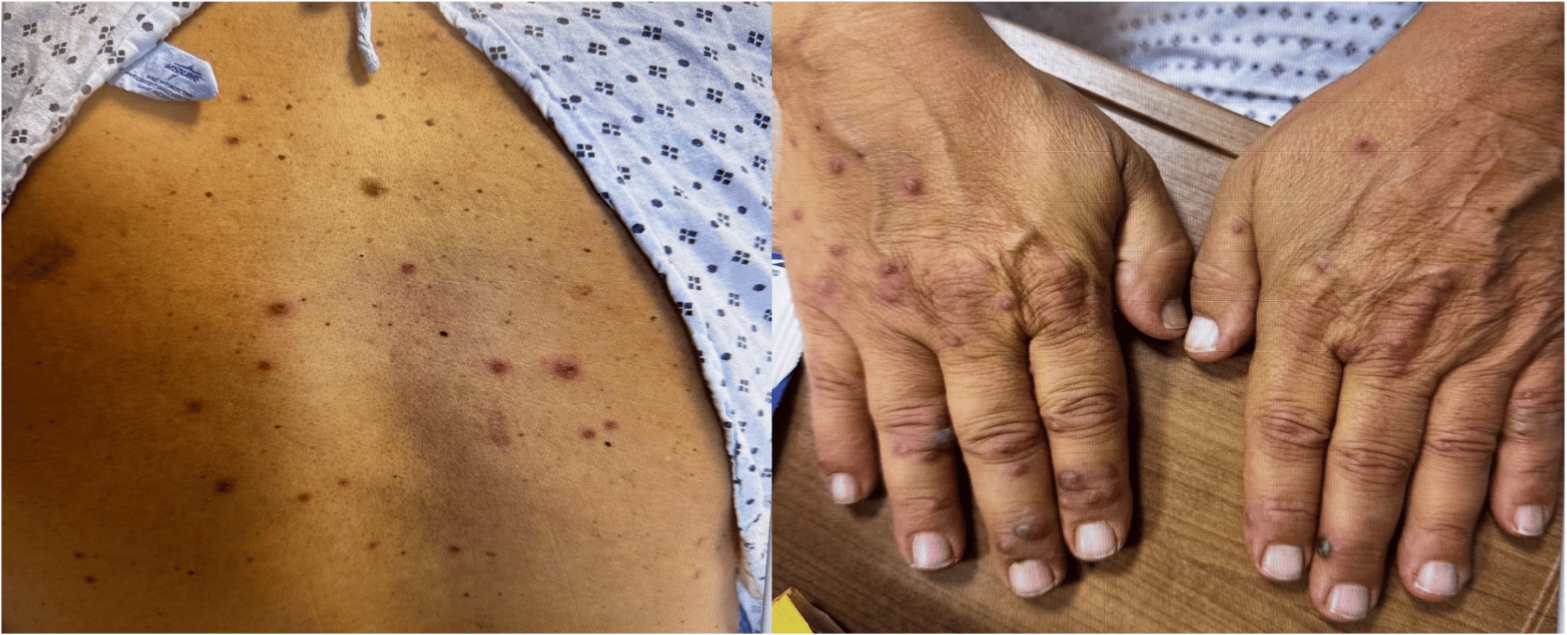
Multiple vesicular lesions observed on the hands and abdomen.

On admission, he was afebrile with a heart rate (HR) of 90 beats per minute, a respiration rate (RR) of 17 breaths per minute, a blood pressure (BP) of 138/90 mmHg, and an oxygen saturation of 98% on room air. Relevant physical examination revealed multiple vesicular lesions on the face, both hands, and posterior trunk without ocular or mucosal involvement (Figure 1). Chest auscultation did not reveal any wheeze or crepitus. There was no palpable hepatomegaly or splenomegaly, and no evidence of icterus was noted. The rest of the general physical and detailed systemic examination, including a complete neurological and cardiovascular evaluation, was unremarkable.

Initial laboratory evaluation revealed significant hepatic and renal dysfunction. Liver function tests showed elevated transaminases with an alanine aminotransferase (ALT) of 1481 units/litre (U/L), aspartate aminotransferase (AST) of 2141 U/L, and alkaline phosphatase (ALP) of 284 U/L. Total bilirubin was 6.9 mg/dL with a direct component of 3.5 mg/dL, and serum albumin was low at 3.1 g/dL. Prothrombin time (PT) and international normalized ratio (INR) were within normal limits (PT: 12.6 seconds; INR: 1.0), indicating preserved hepatic synthetic function. Furthermore, his renal function was deranged, with serum creatinine rising from a baseline value of 1.1 mg/dL to 3.58 mg/dL. The electrolyte abnormalities were also present, including moderate hyponatremia (sodium 125 mmol/L) and hypokalemia (potassium 3.0 mmol/L). Additionally, his serum cortisol levels were markedly elevated at 41.7 μg/dL, likely reflecting a stress response to the systemic infection. Serial laboratory testing demonstrated progressive improvement with antiviral therapy and supportive care (Table 1).

**Table 1:** Serial measurements of liver function tests and creatinine levels before and after the treatment with acyclovir. ALT: alanine aminotransferases; AST: aspartate aminotransferases; ALP: alkaline phosphatase; TB: total bilirubin; DB: direct bilirubin; BUN: blood urea nitrogen

Parameters	Units	Hospital Day 1	Hospital Day 2	Hospital Day 3	Hospital Day 4	Day 12 (follow-up)
ALT	unit/L	1481	786	639	444	48
AST	unit/L	2141	685	286	141	29
ALP	unit/L	284	367	391	329	160
TB	unit/L	6.9	3.8	3.3	2.5	1.4
DB	unit/L	3.5	3.0	2.5	1.8	0.7
Total protein	unit/L	6.3	6.2	6.9	6.4	6.8
Albumin	mg/dL	3.1	3.2	3.5	3.1	3.9
Serum creatinine	mg/dL	3.01	3.58	3.31	2.80	1.5
BUN	mg/dL	35	39	40	29	25

**Figure 2:**
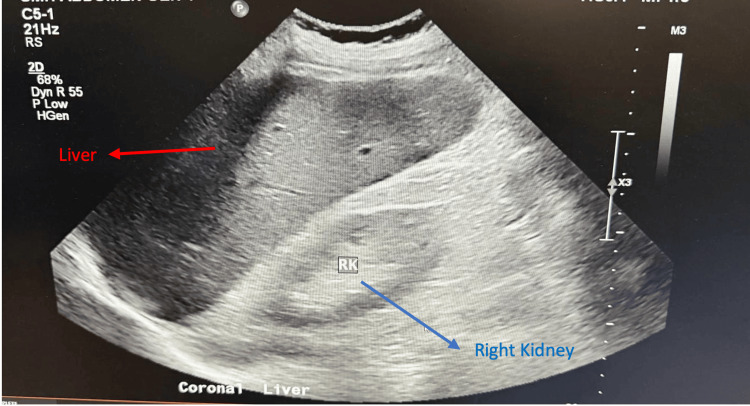
Ultrasound abdomen showing mild hepatosplenomegaly with no evidence of bile obstruction.

An abdominal ultrasound was performed on admission to rule out obstructive causes of hepatitis. It showed mild hepatosplenomegaly without evidence of any vascular stenosis, thrombosis, or biliary duct obstruction (Figure 2).

**Figure 3:**
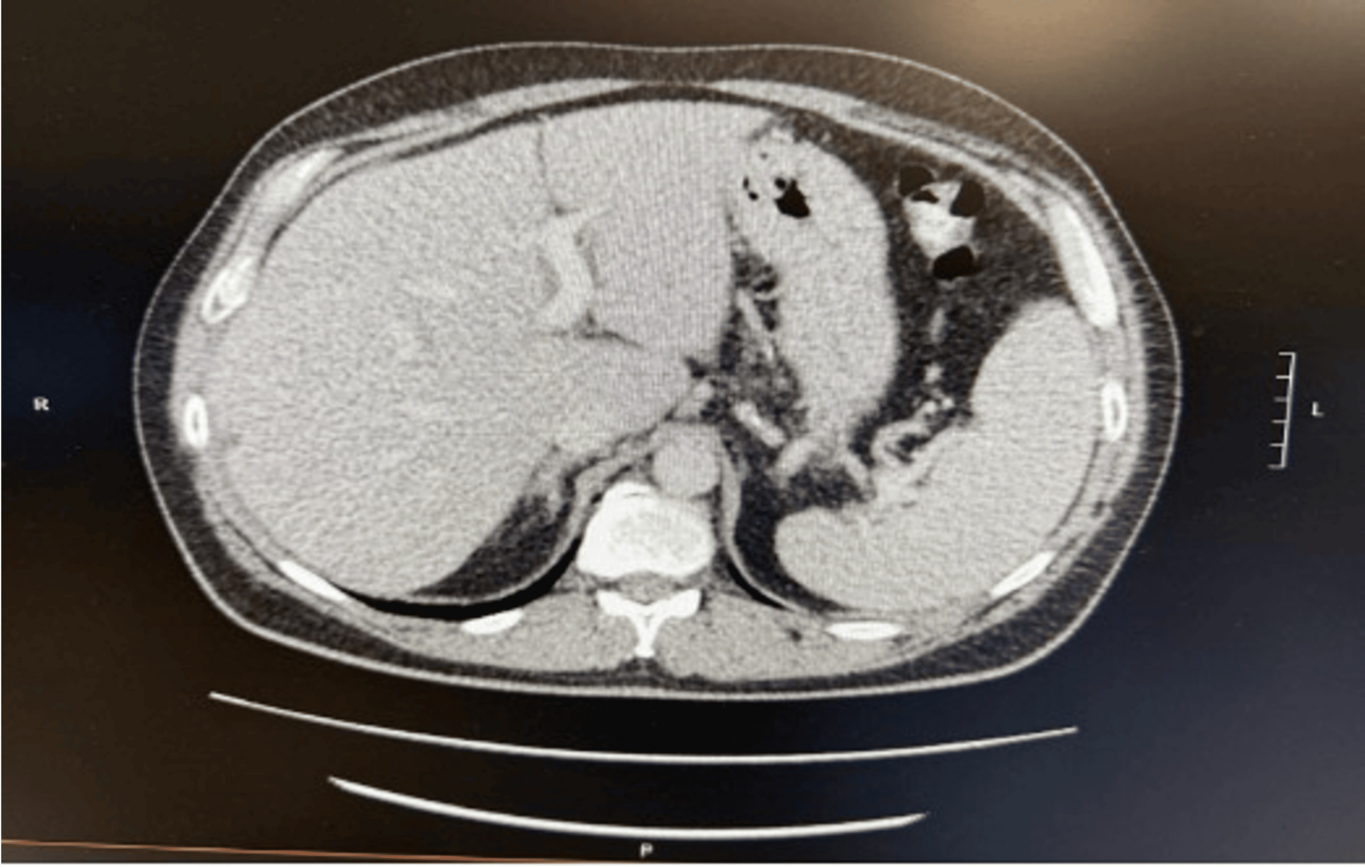
High-resolution contrast-enhanced CT scan of the abdomen (axial view) showing normal liver, gallbladder, pancreas, and spleen.

On hospital day 2, the patient developed abdominal pain, which led to further investigation. A contrast-enhanced abdominal computed tomography (CT) scan was performed, which showed no abnormalities in the liver, gallbladder, pancreas, spleen, adrenals, or kidneys. There was no evidence of renal calculi, obstruction, or intra-abdominal collections (Figure 3).

Given the presence of a diffuse vesicular rash and elevated transaminases, disseminated HSV infection was suspected. Acyclovir was initiated immediately, pending the results of relevant investigations.. Further infectious workup, including tests for Epstein-Barr virus (EBV), cytomegalovirus (CMV), and varicella-zoster virus (VZV), was conducted to rule out other possible viral infections. Likewise, human immunodeficiency virus (HIV) screening, antibiotics, and HIV polymerase chain reaction (PCR) were also performed.. Syphilis screening antibody (Treponema pallidum antibody) was done to detect current or past syphilis. An acute viral hepatitis panel (hepatitis A virus (HAV) IgM, hepatitis B virus (HBV) core IgM, hepatitis B surface antigen (HBsAg), hepatitis C virus (HCV) antibodies) was also sent. Autoimmune serologies, including antinuclear antibodies (ANA) and anti-smooth muscle antibodies (anti-SMA), were also performed to investigate any ongoing autoimmune etiology as a differential. Subsequently, all these investigations came out negative; however, PCR testing for HSV-2 returned positive in both serum and skin lesion swabs.

The diagnosis of disseminated HSV-2 infection with hepatic and renal involvement was established. The patient was continued on intravenous (IV) acyclovir (5mg/kg/dose every eight hours), with careful monitoring of renal and liver functions, and later transitioned to oral valacyclovir (1 g twice daily), completing a 14-day antiviral course. Supportive care, including IV hydration, was provided for acute kidney injury (AKI), which gradually improved (Table 1). The patient was discharged from the hospital in stable condition after a five-day inpatient admission.

At outpatient follow-up three weeks after hospital discharge, all skin lesions had resolved without scarring. Repeat laboratory tests demonstrated normalization of liver and renal function. Due to ongoing high-risk sexual behaviour, the patient was also advised long-acting injectable HIV pre-exposure prophylaxis (PrEP) with lenacapavir, which is administered every six months. He was also counselled on safe sexual practices, and a follow-up visit was scheduled for six months later.

## Discussion

HSV hepatitis is, although rare, a potentially life-threatening condition, typically linked to weakened immune system (immunocompromised states) such as HIV infection, organ transplantation, pregnancy, or the use of immunosuppressive agents such as corticosteroid medications and systemic chemotherapy. Disseminated HSV-2 infection in the immunocompetent population is very rare. However, a literature review by Norvell et al. identified 29 cases of HSV hepatitis in immunocompetent adults between 1986 and 2006, highlighting that severe manifestations of HSV can occur even in patients without apparent immune compromise [3]. In our case, no primary immunodeficiency was noted, and a complete autoimmune and viral workup was negative.

HSV-2 remains among the common sexually transmitted diseases in the United States. Primary infection occurs when an individual is exposed to another infected person who is actively shedding the virus from body secretions or skin [6]. In an immunocompetent host, the pathophysiology that leads to disseminated HSV is unclear. However, possible mechanisms have been reported in the literature, such as initial viremia with the infection that suppresses host immune defences, underlying deficiencies in T-cell and macrophage processing of HSV antigen, superinfection, and variability among HSV strains [7,8]. Hepatic involvement is believed to result from direct viral cytopathic injury to hepatocytes secondary to viremia and immune-mediated hepatocellular necrosis. In contrast, renal involvement may occur through a combination of factors such as systemic inflammatory response, hemodynamic instability leading to pre-renal azotemia, and potential direct viral invasion of renal parenchyma as reported in rare cases. Our case is unique for multiple reasons, such as hepatic and renal involvement, the absence of known immunodeficiency, and a favourable clinical response to antiviral therapy. Importantly, the patient had no prior risk factors for dissemination, underscoring that HSV hepatitis should not be excluded solely based on immune status.

The diagnosis of HSV hepatitis in immunocompetent individuals is often delayed due to its unspecified and rare presentation, which may include fever, abdominal pain, nausea, fatigue, and deranged liver enzymes without overt mucocutaneous lesions. This contributes to the high reported mortality rate, especially when the treatment is not initiated in a timely and appropriate manner. Similar cases in the literature further emphasize the importance of early recognition and empirical antiviral therapy to improve outcomes [3-5,9,10]. In our patient, early clinical suspicion and timely administration of intravenous acyclovir were associated with the patient’s subsequent improvement in the clinical condition, supporting this approach.

Acute renal injury in this case may have been multifactorial and potentially attributable to dehydration, prerenal azotemia from hypovolemia, or direct viral involvement. Although a liver biopsy was not performed to confirm hepatic HSV involvement, the diagnosis was supported by characteristic vesicular skin lesions, radiological findings, and markedly elevated transaminases [11]. An additional consideration in our case is the elevated cortisol level, which may reflect a physiological stress response to severe systemic infection. While not diagnostic, this laboratory finding is consistent with the literature on stress-induced hormonal changes in critical illness.

The patient was also advised long-acting HIV PrEP with lenacapavir, a relatively novel agent. It is a first-in-class capsid inhibitor that is administered subcutaneously (SC) every six months, providing a convenient alternative to daily oral PrEP. Recent studies suggest this long-acting formulation may improve adherence and reduce breakthrough infections in high-risk populations [12]. In patients with HSV-2 infection, it is critical to recognize and address the associated risk factors, such as multiple sexual partners, unprotected sexual activity, and a detailed history of other sexually transmitted infections. It is also essential to educate affected individuals that HSV-2 infection not only increases the risk of recurrent outbreaks but also facilitates transmission of HIV and other sexually transmitted diseases (STDs). Patient counselling plays a vital role in prevention, further emphasizing the regular use of barrier protection, disclosure to partners, and regular sexual health check-ups, hence, maintaining long-term sexual and reproductive health.

## Conclusions

This case contributes to the growing body of literature highlighting that disseminated HSV, including hepatitis, should be considered in the differential diagnosis of vesicular rash with hepatic dysfunction, even in immunocompetent individuals. Early clinical suspicion and initiation of antiviral therapy are crucial to improving health outcomes in this often-overlooked diagnosis. Clinicians should remain vigilant for atypical presentations to ensure timely diagnosis and prevent potentially life-threatening complications.
